# *In situ* molecular identification of the Influenza A (H1N1) 2009 Neuraminidase in patients with severe and fatal infections during a pandemic in Mexico City

**DOI:** 10.1186/1471-2334-13-20

**Published:** 2013-01-18

**Authors:** Rodolfo Ocadiz-Delgado, Martha Estela Albino-Sanchez, Enrique Garcia-Villa, Maria Guadalupe Aguilar-Gonzalez, Carlos Cabello, Dora Rosete, Fidencio Mejia, Maria Eugenia Manjarrez-Zavala, Carmen Ondarza-Aguilera, Rosa Ma Rivera-Rosales, Patricio Gariglio

**Affiliations:** 1 Department of Genetics and Molecular Biology, CINVESTAV-IPN, Mexico City, Mexico; 2 Department of Virology and Micology Research, Instituto Nacional de Enfermedades Respiratorias Ismael Cosio Villegas, Calz. De Tlalpan 4502, Colonia Sección XVI, CP 14080, Mexico, DF, Mexico; 3 Department of Pathoplogy, Instituto Nacional de Enfermedades Respiratorias Ismael Cosio Villegas, Mexico City, Mexico; 4 Clínic 32, Instituto Mexicano del Seguro Social, Mexico City, Mexico

**Keywords:** Influenza virus, *In situ* RT-PCR, Influenza diagnosis, Influenza pneumonia, Influenza pandemic in Mexico City

## Abstract

**Background:**

In April 2009, public health surveillance detected an increased number of influenza-like illnesses in Mexico City’s hospitals. The etiological agent was subsequently determined to be a spread of a worldwide novel influenza A (H1N1) triple reassortant. The purpose of the present study was to demonstrate that molecular detection of pandemic influenza A (H1N1) 2009 strains is possible in archival material such as paraffin-embedded lung samples.

**Methods:**

In order to detect A (H1N1) virus sequences in archived biological samples, eight paraffin-embedded lung samples from patients who died of pneumonia and respiratory failure were tested for influenza A (H1N1) Neuraminidase (NA) RNA using *in situ* RT-PCR.

**Results:**

We detected NA transcripts in 100% of the previously diagnosed A (H1N1)-positive samples as a cytoplasmic signal. No expression was detected by *in situ* RT-PCR in two Influenza-like Illness A (H1N1)-negative patients using standard protocols nor in a non-related cervical cell line. *In situ* relative transcription levels correlated with those obtained when *in vitro* RT-PCR assays were performed. Partial sequences of the NA gene from A (H1N1)-positive patients were obtained by the *in situ* RT-PCR-sequencing method. Sequence analysis showed 98% similarity with influenza viruses reported previously in other places.

**Conclusions:**

We have successfully amplified specific influenza A (H1N1) NA sequences using stored clinical material; results suggest that this strategy could be useful when clinical RNA samples are quantity limited, or when poor quality is obtained. Here, we provide a very sensitive method that specifically detects the neuraminidase viral RNA in lung samples from patients who died from pneumonia caused by Influenza A (H1N1) outbreak in Mexico City.

## Background

During April 2009, the number of atypical pneumonia cases increased in Mexico City’s hospitals and spread to almost all boroughs in the city; these cases were related to a new influenza A (H1N1) virus strain that was identified as the etiological agent [1-4]. In less than a month the virus spread worldwide and on June 11, 2009 the World Health Organization (WHO) declared the start of the first 21st century influenza pandemic.

Influenza A viruses belong to the *Orthomyxoviridae* family; they are characterized by a unique genome structure with a single-negative RNA strand, which codifies, among others, for two transmembrane proteins: hemagglutinin (HA) and neuraminidase (NA) [5-7]. HA plays an important role during the cell entry of influenza viruses. This protein is essential during the initial steps of infection because it is responsible for the attachment of the virus to sialic acid (SA) cellular receptor. This interaction explains, at least in part, the host range and tissue tropism of influenza viruses [5,8]. The NA of influenza viruses is a homotetrameric glycoprotein anchored by a fibrous stalk in the viral membrane. The protein possesses a globular head comprised of four monomers that constitute the active site composed of nine conserved residues. Its primary role in the infectious cycle is to liberate the viral progeny from infected cells. Its enzymatic activity catalyzes SA removal from its linkage to galactose, thereby destroying the receptor and allowing the virus to disseminate and infect other cells [8,9]. Furthermore, NA is also the main target of the antiviral drugs zanamivir and oseltamivir. These drugs closely resemble the structure of the natural substrate of the NA and thus prevent the removal of the SA residue from the glycopeptide receptor by the viral neuraminidase [5]. In addition to the increased transmissibility and the low or lack of immunity of the human population, the fact that new reassortment events may alter the pathogenicity of circulating strains, makes it crucial to monitor the progress of the pandemics at the molecular level [2,10,11].

Molecular methods are becoming more widely used for the detection of respiratory pathogens, in part because of their superior sensitivity, relatively rapid turnaround time, and ability to identify pathogens that are slow growing or difficult to culture. The recent novel H1N1 influenza A pandemic has been useful to underscore how quickly new molecular tests can become available for clinical use [12]. Previously, several groups have been used immunohistochemical or immunofluorescence detection for determine the localization of influenza virus antigens, as well as *in situ* hybridization for detection of viral sequences and ultrastructural examination to detect viral particles, in cases of fatal H1N1 influenza A virus infection during the period 2009–2010 [13-19]. These analyses were performed on sections from different tissues such as respiratory tissues (trachea, lung), heart, liver, and placenta [13-19]. Over the years, Reverse-transcriptase PCR is the recommended test for diagnosis and confirmation of infections due to pandemic 2009 influenza A(H1N1) virus [20]. Recently, modifications of this technology have emerged, some of which allow the rapid detection of multiple pathogens in a single test such as multiplex molecular technologies, reverse transcriptase-PCR, real-time PCR, microarrays and nucleic acid sequencing-based amplifications [12,21,22]. Other studies have also shown the usefulness of rapid immunoassays for seasonal influenza virus [23]. These methods have greatly enhanced the capability for surveillance and characterization of influenza viruses and their clinical utility for the detection of respiratory pathogens. However, these methods can not be easily applied for the analysis of paraffin-embedded tissues. *In situ* RT-PCR has some major strengths for the detection of specific nucleic acid sequences. First and foremost, one can detect particular sequences on archival material; second, this technique combines the extreme sensitivity of PCR with the cell localization ability similar to *in situ* hybridization [17]. A third strength of *in situ* RT-PCR relates to the issue of sample contamination in solution-phase PCR. Sample contamination, which can lead to false-positive results in PCR, limits its value as a diagnostic test for viral infections; this limitation is not encountered in *in situ* RT-PCR. Fourth, this technique is the only amplification technique that allows direct target-specific incorporation of a reporter nucleotide (such as DIG-digoxigenin-dUTP-labeled nucleotide) [24,25], thus eliminating the need for a hybridization step. Clearly, *in situ* RT-PCR has been useful for any target that is low copy and, thus, difficult to detect with standard *in situ* hybridization, which has a detection threshold of 10 to 20 copies per cell [17].

The purpose of the present study was to demonstrate that sensitive and specific molecular detection of pandemic influenza A (H1N1) 2009 strains is possible in archival material such as paraffin-embedded lung samples. This strategy would be useful to perform current and retrospective studies in a specific and reproducible manner.

## Methods

### Autopsy material

This project was approved after being checked by the “Institute Science and Bioethics Committee” (INER-Mexico). The Committee is responsible for evaluating the research projects to be performed, in order to monitor that every study meets the principles for research involving human subjects, established in the Declaration of Helsinki and its different revisions. The approval number is: B07-09.

From April 2009 to February 2010, eight lung samples were taken *post mortem* from patients who died of pneumonia and respiratory failure at Instituto Nacional de Enfermedades Respiratorias-Ismael Cosío Villegas (INER) and Clinica-32 of Instituto Mexicano del Seguro Social (IMSS), Mexico City. At least three autopsy samples were collected mainly from medium upper lobe of the lung (Table 1). The size of lung autopsy samples ranged from 20 to 80 mm^2^ area. Lung samples were paraffin-embedded, sectioned to obtain 5 μm thick sections and mounted on electrostatically charged slides. Molecular procedures (RT-PCR, *in situ* RT-PCR, sequencing and *in vitro* duplex amplification technique) were performed in all 8 patient lung samples, as well as in control samples. All specimens were studied by an expert pathologist (R.M.R.R.).

**Table 1:** List of autopsy samples obtained from A H1N1 positive patients

***Patient***	***Location of sample***	***Size of sample(Average;mm***^***2***^**)**	***Number of samples taken***
P01	Central and peripheral	30	3
P02	Central	24	4
P03	Central	40	3
P04	Central and peripheral	20	4
P05	Central	24	3
P06	Central	80	3
P07	Central and peripheral	66	2
P08	Central and peripheral	72	3

### Molecular detection of A (H1N1) viruses

The detection of pandemic influenza A (H1N1) 2009 viruses was done using RT-PCR standard protocols as part of the Virology Department’s surveillance routine and has been described elsewhere (http://www.who.int/csr/resources/publications/swineflu/realtimeptpcr/en/index.html; [2,26]). All specimens were accompanied by a standard form with information about age, sex, date of illness onset, date of specimen collection, place of residence, clinical features of each patient, travel history, vaccination history and administration of antiviral treatment.

### Primers

Neuraminidase (NA) specific primer sequences were selected according to the need of the *in situ* technique: NA/AH1N1F (sense) 5’-ACCATTGGTTCGGTCTGTATG-3’ and NA/AH1N1R (antisense): 5’-GAGGCCTGTCCATTACTTGGPU-3’. These primers have been pre-validated by liquid-phase RT-PCR method that allows the detection of a 729 base pairs (bp) band in agarose gel electrophoresis on clinical samples obtained from patients infected with A (H1N1) (Manjarrez-Zavala *et al*., manuscript in preparation). As internal constitutive expression control, β2-microglobulin (β2-m) transcripts were detected using the following primers: β2mF (sense): 5’-ACCCCCACTGAAAAAGATGAGTAT-3’ and β2mR (antisense): 5′-ATGATGCTGCTTACATGTCTCGAT-3′ [27-29]. β2-m PCR size product was of 100 bp. All primers were purchased from Invitrogen (U.S.A.).

### *In situ* RT-PCR

**Figure 1:**
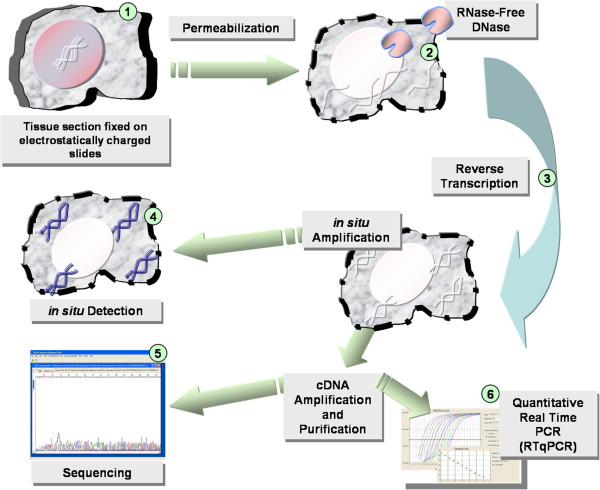
**Experimental design used to detect the Neuraminidase**-**gene derived nucleic acid of A (H1N1) virus by *in situ *RT**-**PCR.** Tissue fixed on electrostatically charged slides (**1**) was permeabilized and genomic DNA was eliminated using RNase-free DNase (**2**). After *in situ* reverse transcription (**3**), synthesized cDNA was amplified and detected on tissue (**4**). Reverse Transcription reaction product was recovered for amplification using *in vitro* PCR and subsequent sequencing (**5**) and quantification by Real Time quantitative PCR (RTqPCR) (**6**).

Direct *in situ* RT-PCR was performed as previously described with some modifications [24,25,30-32] (Figure 1). Briefly, dried dewaxed sections on electrostatically charged slides were incubated with protein lysis buffer (0.1 M Tris–HCl pH 8.0, 50 mM EDTA pH 8.0) containing 0.5 μg/ml Proteinase K for 30 min at room temperature. After Proteinase K digestion, each tissue section was treated with 50 μl of a solution containing 1 U of DNase I, RNase-free (Roche, U.S.A.) during 48 h at room temperature. After thoroughly washing with DEPC-treated water, *in situ* reverse transcription was performed using the SuperScript II reverse transcriptase (Invitrogen, U.S.A.), following the manufacturer’s specifications. In brief, 70 μl DEPC-treated water containing 3 μg of random primers oligonucleotides (mostly hexamers; Invitrogen, U.S.A.), 10 mM dNTP mix (10 mM each dATP, dGTP, dCTP and dTTP at neutral pH), 5X first-strand buffer, 10 mM DTT, recombinant ribonuclease inhibitor (40 U/μl) and reverse transcriptase (100 U/ tissue section) (Invitrogen, U.S.A.) were added to each section. Slides were incubated at 42°C for 1 h in a sealed humidified chamber. After thoroughly washing with ultrapure water, 50 μl of the PCR master mix solution containing 100mM digoxigenin-11-(2'-deoxy-uridine-5')-triphosphate (DIG-11-dUTP; Roche, U.S.A.), 10X PCR buffer, 50 mM MgCl_2_ and primer mix (10 μM each of forward and reverse primers) were added [31]. To reduce primer-dimer formation the PCR solution was heated to 70°C for 10 min before Taq DNA polymerase (5 U per reaction) was added. Negative controls included tissue obtained from two Influenza-like Illness (ILI) patients that were negative for the influenza A (H1N1) virus. These A (H1N1) negative patients showed: (1) severe acute respiratory illness defined as dyspnea plus bilateral infiltrates on the x-ray; (2) lack of 2009 H1N1 infection; and (3) a stay of at least 24 h in the intensive care units. Samples were taken after patients died. In addition, reactions without primers or reactions without Taq DNA polymerase were performed in consecutive tissue sections from all samples. *In situ* PCR was performed using the system provided by Perkin Elmer (U.S.A.). Fifty microliters of PCR master mix were added to each sample and the reaction was sealed using AmpliCover discs and clips (Perkin Elmer, U.S.A.). After assembly, slides were placed at 70°C in the GeneAmp *In situ* PCR system 1000 (Perkin Elmer, U.S.A.) until running was started (18 cycles). Amplifications for NA or β2-m were performed separately. Samples were first heated to 94°C (3 min) and then subjected to 18 cycles of: 94°C/ 1 min, 60°C/ 1.5 min and 72°C/ 1 min. After amplification a 10-min elongation step at 72°C was carried out. After cycling was complete, the temperature was kept at 4°C until disassembly. Clips were removed and AmpliCover discs were very carefully lifted from the slides without moving them sideways and slides were washed for 5 min in PBS followed by 5 min in 100% EtOH before they were air dried [30]. To ensure consistency and reproducibility and to eliminate PCR artifacts, all assays were performed on a minimum of three separate occasions. Slides were processed in order to detect *in situ* PCR products (see below).

### Detection of *in situ* PCR products

An indirect immunolabeling method using a primary Anti-Digoxigenin antibody (Fab fragments; Roche, U.S.A.) conjugated to alkaline phosphatase was chosen to detect the PCR product. Briefly, blocking was carried out in 5% BSA (Sigma, U.S.A.) in PBS for 30 min. Slides were then drained and incubated with Anti-DIG antibody diluted 1:200 in 100 mM Tris–HCl pH 7.4 and 150 mM NaCl (100 μl per sample) for 2 h at room temperature. As negative control the primary antibody was omitted. Detection of alkaline phosphatase was carried out for 10–30 min using NBT/BCIP kit (Zymed, U.S.A.). After detection, slides were rinsed in distilled water for 5 min and air dried before mounting in Permount histological mounting medium (Fisher Scientific, U.S.A.).

### Digital Image Capture, analysis and quantification

All photomicrographs were obtained using a DFC290 HD digital camera (Leica Microsystems, USA). The following method of quantification was used for *in situ* RT-PCR analyses. After acquisition of the images using the digital camera, the experimental image files were opened in the PhotoImpact software (Ulead PhotoImpact SE version 3.02; Ulead Systems, U.S.A.). The images were digitally processed in order to obtain the better and homogeneous signal and then selected for analysis of relevant regions. The selected regions were then digitally analyzed using the Image-ProPlus Analysis Software (Ver 4.5.0.19, Media Cybernetics, Inc., U.S.A.). The amount of signal was quantified using a pixel matrix data (the color contained within a pixel inside an image at specific location). Therefore, chromogen quantity was determined by calculating the norm of the matrix file for that image. This allows pixels of similar “color” immediately adjacent to the index pixel to be included for analysis. All pixels falling within the selected threshold parameters were quantified, recorded and used to generate a graphic. The file for the control image was similarly generated: the control slide is acquired and treated identically as the experimental slide. Negative controls included: Two A (H1N1)-negative samples, reactions without reverse transcriptase and reactions without anti-DIG antibody. Beta2-microglobulin signal was used to normalize data in case of *in situ* RT-PCR quantification.

### Sequencing of *in situ* RT-PCR products

After *in situ* reverse transcription, solution over the tissue section was recovered and the cDNA contained in this solution was amplified using standard liquid-phase PCR. The nucleotide sequences of the PCR products were determined using the BigDye Terminator v 3.1 Cycle sequencing kit (Applied Biosystems), purified using the ZR DNA Sequencing Clean-up Kit (Zymo Research Corp. U.S.A.) and analyzed on the ABI Prism 310 Analyzer Sequencer (Applied Biosystems). The obtained sequences were aligned and analyzed using Basic Local Alignment Search Tool (BLAST; http://blast.ncbi.nlm.nih.gov). Sequences obtained from amplification corresponded to the segment between the middle part and the 3’ end of the NA gene (Figure 1). We have included two positive controls: a Puerto Rico/Puerto Rico/IvPR8/Puerto Rico laboratory strain and an A (H1N1) positive sample obtained from a patient with Influenza A virus A/reassortant/NYMC X-179 (California/07/2009 × NYMC X-157) (H1N1) (GenBank: CY058512.1) (Tables 2 and 3).

**Table 2:** Clinical data of influenza illness patients

***Patient***	***Fever***	***Cough***	***Headache***	***Sore throat***	***Rinorrhea***	***Myalgia***	***Chills***	***Nasal Congestion***	***Conjunctivitis***	***A(H1N1)Conventional Methods(RT***-***PCR standard protocols)***	***A(H1N1)in situ RT***-***PCR***
P01	+	+	+	+	-	+	+	+	+	+	+
P02	+	+	+	+	+	-	+	-	-	+	+
P03	+	+	-	-	+	+	-	-	-	+	+
P04	+	+	+	+	+	+	+	-	-	+	+
P05	+	-	+	-	-	-	-	+	+	+	+
P06	+	+	+	-	+	+	-	-	-	+	+
P07	+	+	+	+	+	-	+	-	-	+	+
P08	+	+	-	+	-	+	-	+	+	+	+
Positive Control 1 [A (H1N1) laboratory strain]	ND	ND	ND	ND	ND	ND	ND	ND	ND	+	+
Positive Control 2 [A (H1N1) positive patient]	**+**	**+**	**+**	**-**	**-**	**+**	**+**	**+**	**+**	**+**	ND
Negative Control 1 (Influenza-negative Patient 1)	-	-	-	-	-	-	-	-	-	-	-
Negative Control 2 (Influenza-negative Patient 2)	-	-	-	-	-	-	-	-	-	-	-
Negative Control (HeLa cervical cell line)	ND	ND	ND	ND	ND	ND	ND	ND	ND	-	-

ND: Not Determined.

**Table 3:** List of Influenza A virus sequences detected in clinical specimens

***Patient***	***Influenza A subtype***	***Isolate name***	***Homology***
P01	A (H1N1)	Ontario/Mexico City/Auckland/Jalna	98%
P02	A (H1N1)	Ontario/Mexico City/Auckland/Jalna	98%
P03	A (H1N1)	Viena/Yaroslavl/Texas	98%
P04	A (H1N1)	Ontario/Mexico City/Auckland/Jalna	98%
P05	A (H1N1)	New York/San Salvador	98%
P06	A (H1N1)	Jalna/Auckland	97%
P07	A (H1N1)	Puerto Rico/IvPR8	96%
P08	A (H1N1)	Texas/Wisconsin/Roma/Colombia District	98%
Positive Control 1	A (H1N1)	Puerto Rico/Puerto Rico/IvPR8/Puerto Rico	98%
Positive Control 2	A (H1N1)	A/reassortant/NYMC X-179 (California/07/2009 x NYMC X-157) (H1N1)	99%

### *In vitro* Duplex amplification technique

*In vitro* duplex amplifications were performed using 2 μL of cDNA as template and reaction mixtures (25 μL) containing: 1x PCR buffer, 1.5 mM of MgCl_2_, 200 μM of each dNTP, 400 nM of each primer, 2.5 units of Taq DNA polymerase (Invitrogen, U.S.A.). Specific primers recognizing NA or β2-microglobulin sequences used for *in situ* amplification were included in the reaction (amplification size products of 729 and 100 base pairs, respectively). Amplifications were carried out using a GeneAmp PCR System 9700 thermal cycler (Applied Biosystems, U.S.A.). The duplex protocol included 3 minutes incubation at 95°C followed by 40 cycles of 1 min at 94°C, 1.5 min at 60°C and 1 min at 72°C. A final extension of 10 minutes at 72°C was performed. Finally, the duplex PCR products were separated by electrophoresis on a 2% agarose gel stained with ethidium bromide and visualized under UV light.

### RNA isolation

As a control for *in situ* determinations, total RNA was isolated from autopsy samples obtained from patients with influenza illness using TRIzol reagent according to the manufacturer’s instructions (Invitrogen). The RNA preparations were used for cDNA synthesis and Real-Time PCR.

### *Preparation of cDNA for Real-Time quantitative PCR (RTqPCR)*

Three micrograms of total RNA were reverse transcribed in a 20 μl reaction using 100 U SuperScript II Reverse Transcriptase, following the manufacturer′s specifications (Invitrogen). In parallel, cDNAs synthesized during the *in situ* reverse transcription reaction were recovered and used for sequencing (Figure 1).

### Relative RNA Quantification by RTqPCR

The relative quantification of the A (H1N1) viral load was determined by RTqPCR using a 7300 Real Time PCR System (Applied Biosystems, USA). PCRs were processed through 35 cycles of a 3-step PCR, including 10 sec of denaturation at 95°C, a 10 sec primer dependent annealing phase (60°C), and a 10 sec template-dependent elongation at 72°C. The amplification of each template was performed in duplicate in one PCR run. The relative viral load was calculated as the ratio normalized to β2-microglobulin.

### RTqPCR data analysis using 2^-ΔΔCT^ method

Real-time PCR was performed on the corresponding cDNA synthesized from each *in situ* sample. The data were analyzed using the equation described by Livak [32]. Validation of the method was performed as previously reported [32,33].

## Results

### Clinical features of patients with Influenza

A total of 8 lung samples obtained from patients who died of pneumonia and respiratory failure influenza-positive patients (3 females and 5 males) were analyzed using *in situ* RT-PCR for influenza A (H1N1) (2009). The most common symptoms among the infected subjects were fever over 39 °C (100%), cough (94%), headache (84%), sore throat (72%), rinorrhea (71%), myalgia (69%), chills (50%), nasal congestion (44%), and conjunctivitis (40%) (Table 2).

### Expression levels of Neuraminidase A (H1N1) RNA in patients infected with Influenza

**Figure 2:**
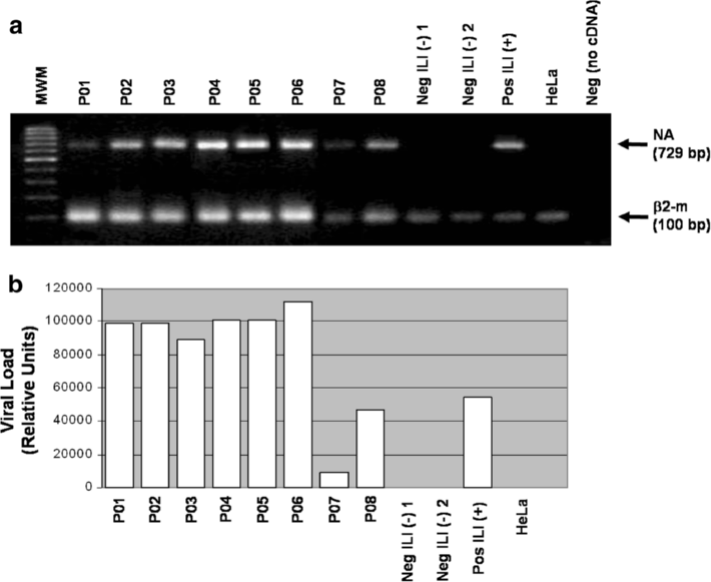
**Neuraminidase A (H1N1) expression levels in clinical samples.** (**a**) Duplex RT-PCR of A (H1N1) Neuraminidase (NA) and β2-microglobulin (β2-m) in the eigth A (H1N1)-positive samples (P01-P08). In all experiments, two negative Influenza-like illness patients [Neg ILI (−) 1, Neg ILI (−) 2] and positive [Pos ILI (+); H1N1 Puerto Rico/Puerto Rico/IvPR8/Puerto Rico; 98% homology] sample were included as controls. In addition, HeLa cervical cell line and a negative control with no sample (no cDNA) were included [HeLa and Neg (no cDNA), respectively]. RNA preparations, obtained from nasopharyngeal swabs of A (H1N1) positive cases were assayed using NA or β2-m specific primers (729 and 100 bp amplification product, respectively). MWM: Molecular weight marker. (**b**) Neuraminidase A (H1N1) expression levels determined in the same patient’s samples by quantitative RTqPCR. Viral load results are expressed as Relative Units.

Specific primers recognizing the NA A (H1N1) influenza gene were used to establish expression levels in RNA samples. NA relative expression levels were determined in all eight samples using the β2-microglobulin housekeeping mRNA as internal control (Figure 2a). Primers and duplex amplification technique were validated analyzing a large number of nasopharyngeal swabs obtained from ILI patients (Manuscript in preparation). In addition, all samples were analyzed by quantitative RTqPCR (Figure 2b).

### NA A (H1N1) influenza gene expression can be detected in autopsy samples using *in situ* RT-PCR

**Figure 3:**
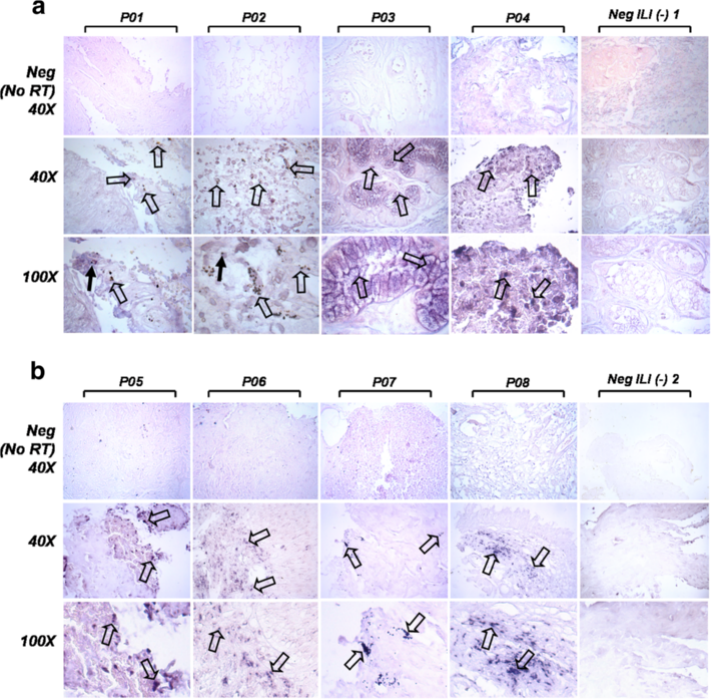
**Representative images of A (H1N1)RNA detection in lung samples obtained from eight patients with influenza**-**like illness and pneumonia using *in situ *RT**-**PCR.** Tissue was processed and *in situ* RT-PCR was done with NA specific primers as described in the Methods section and in Figure 1. Arrows indicate intense cytoplasmic signal in P01-P04 (a) and P05-P08 (b) positive samples in comparison with samples obtained from two A (H1N1)-negative patients [Neg ILI (−) 1 and 2]. Obtained signal suggested that amplification was positive in pneumocytes (black empty arrows) or macrophage-like cells (solid arrows; patients P01 and P02) and tracheal epithelium (patient P03). Recovered cDNA was subsequently amplified and PCR products were validated using sequencing reactions. (**a**) P01-P04: Tissue samples from patients 1 to 4. Neg ILI (−) 1: Negative Influenza-like illness patient 1. (**b**) P05-P08: Tissue samples from patients 5 to 8. Neg ILI (−) 2: Negative Influenza-like illness patient 2. Neg (No RT): Negative controls without reverse transcription reaction. Magnification: 40X and 100X.

The expression pattern of the NA viral gene in lung samples obtained from patients with pneumonia was determined by *in situ* RT-PCR. We detected the expression of the NA gene in 100% of the samples (Figure 3; Table 2). As expected, no expression was detected by *in situ* RT-PCR in two A (H1N1)-negative patient samples nor in non-related cells (HeLa cervical cancer cell line; Figure 3). Relative *in situ* transcription levels correlated with those obtained when *in vitro* RT-PCR assays were performed. Interestingly, the *in situ* signal localization suggests that A (H1N1) influenza virus infects alveolar-like cells.

### Sample sequencing

Partial sequences of NA gene from A (H1N1)-positive patients were obtained by the *in situ* RT-PCR-sequencing method (Table 3). In all experiments, negative Influenza-like illness patients [Neg ILI (−)] and two positive [H1N1 Puerto Rico/Puerto Rico/IvPR8/Puerto Rico; 98% homology and A/reassortant/NYMC X-179(California/07/2009 × NYMC X-157) (H1N1), GenBank: CY058512.1, 99%] controls were included (Tables 2 and 3). In order to avoid contamination and subsequent positive false results, we have performed sequence determinations at least in triplicate and we have also included a negative control without template. Sequence analysis showed 98% similarity with influenza viruses reported previously in places such as New York/San Salvador (98% homology; one of eight patients); Texas/Wisconsin/Roma/Colombia District (98% homology; one patient); Ontario/Mexico City/Auckland/Jalna (98% homology; 3 patients); Viena/Yaroslavl/Texas (98% homology; one patient). Lower sequence homology was determined with Jalna/Auckland (97% homology; one patient) and Puerto Rico/IvPR8 (96% homology; one patient).

## Discussion

In this study we describe a successful method for molecular detection of A (H1N1) influenza virus RNA in lung sections obtained from persons who died during the epidemic outbreak of influenza A (H1N1) 2009 in Mexico City. Although the current diagnostic technologies are reasonably effective for sporadic and epidemic influenza [34], new strategies including more sensitive and more specific tests have been developed enhancing not only diagnostic capability but also the need for retrospective testing [34]. The main goal of this work was the standardization of viral RNA genome detection and viral load determination in patients where sample collection such as good-quality RNA could not be performed.

In order to demonstrate that A (H1N1) Neuraminidase transcript was expressed in infected samples, we decided to use *in situ* RT-PCR. This technique combines the extreme sensitivity of PCR with the cell localizing ability similar to *in situ* hybridization and allows comparing epithelia with stroma. *In situ* RT-PCR results demonstrated that A (H1N1) influenza virus could be detected in the same tissue as that in which histopathological lesions had been observed (Figure 3). Furthermore, sequencing data confirmed that *in situ* technique was capable to specifically detect viral sequences. Although the primary goal of this study was to validate the *in situ* RT-PCR detection of NA influenza viral gene, we thought that these data may suggest that Mexico City could have played an important role for the dissemination of some variants throughout the world as indicated by the match between the Mexican samples included in this study and sequences described in other Countries such as New York, San Salvador, Texas, Wisconsin, Roma, Colombia District, Ontario, Auckland and Puerto Rico. In order to establish a phylogenetic analysis, the entire genome from the viruses should ideally be sequenced (Neuraminidase gene contains approximately 1,413 bp); the partial NA sequences generated by our group (729 bp) are of importance as they could provide a baseline of NA sequences from where the pandemic emerged. Additional phylogenetic analyses would be necessary to provide a better understanding of influenza NA lineages and their evolutionary dynamics, which may facilitate early detection of newly emerging influenza viruses and thus improve influenza surveillance and public health [35].

Recently, immuno-histopathological analyses of lung autopsy lung from patients with viral pneumonia revealed that the alveolar epitheliums as well as the alveolar macrophages are key target cells infected by influenza [36-42]. Similar conclusions have also been drawn from experiments where *ex vivo* lung tissue has been infected with the H5N1 virus [43-45]. Interestingly, morphological analyses performed in this study suggest that the *in situ* signal was detected in alveolar macrophage-like cells; however, the *in situ* detection of additional biomarkers would be necessary to provide evidence of the cellular type that is susceptible to A (H1N1) viral infection (i.e. bronchial and bronchiolar epithelial cells, pneumocytes and/or macrophages).

Molecular strategies such as *in situ* hybridization (ISH) have been used to identify cells infected with influenza virus in lung and other tissues [46-49]. One of the main features of the *in situ* RT-PCR is to take advantage of the *Taq* DNA polymerase to amplify a very low number of copies of a specific sequence in comparison with ISH in which the results depend on one-to-one strand recognition. To the best of our knowledge, the *in situ* RT-PCR strategy has been used in a limited number of cases for detecting influenza viral sequences in tissues such as myocardium and placenta [18,50]. Therefore, this is the first time that *in situ* detection of A H1N1 NA sequences is performed in lung sections. One novel advantage that we have developed performing *in situ* RT-PCR is the ability of recover and subsequently to amplify the cDNA synthesized during the *in situ* reverse transcription reaction, this cDNA can be used to perform nucleic acid sequence analyses as well as to detect novel biomarkers.

## Conclusion

In summary, *in situ* retrospective detection of influenza A (H1N1) virus arising in Mexico City could provide important information to study the natural viral spread of influenza virus and, if it is possible, to correlate these data with the emergence of future new pathogenic influenza virus pandemic. Although the majority of pathology laboratories do not perform *in situ* RT-PCR techniques, the development of accurate tests for the detection of influenza in archival paraffin-embedded samples could be a complementary tool for the detection of the virus, enabling the laboratory to provide a prompt, definitive diagnosis, which would allow clinicians to initiate preventive methods, implement appropriate infection-control measures eventually decreasing the incidence of influenza cases, diminishing duration of hospitalization, reducing ancillary testing, and decreasing health care costs.

## Competing interests

The authors declare they do not have conflict of interest.

## Authors’ contributions

ROD. Participated in the study design, coordination and draft the manuscript; Carried out and interpretation the molecular studies. MEAS and EGV. Participated in sequence alignment and drafted the manuscript. MGAG. Participated in sample sequencing and patient data case studies. CC. Participated in the clinic studies and patient data case studies. DR. Carried out and interpretation the molecular studies. FM. Assistance on characterization of the isolates with reference strains. MEMZ. Conceived the study and participated in its design and draft the manuscript. Participated in clinic and case studies. COA. Obtained the samples and performed the data analysis. RMRR. Take autopsy samples and performs the pathological study. PG. Conceived the study and participated in its design and draft the manuscript. All authors have read and approved the final manuscript.

## Pre-publication history

The pre-publication history for this paper can be accessed here:

http://www.biomedcentral.com/1471-2334/13/20/prepub
